# Altered Tregs Differentiation and Impaired Autophagy Correlate to Atherosclerotic Disease

**DOI:** 10.3389/fimmu.2020.00350

**Published:** 2020-03-13

**Authors:** Sara Mandatori, Ilenia Pacella, Vincenzo Marzolla, Caterina Mammi, Donatella Starace, Fabrizio Padula, Laura Vitiello, Andrea Armani, Carmine Savoia, Maurizio Taurino, Daniela De Zio, Claudia Giampietri, Silvia Piconese, Francesco Cecconi, Massimiliano Caprio, Antonio Filippini

**Affiliations:** ^1^Unit of Histology and Medical Embryology, Department of Anatomy, Histology, Forensic Medicine and Orthopaedics, Sapienza University of Rome, Rome, Italy; ^2^Laboratory of Cellular and Molecular Immunology, Department of Internal Clinical Sciences, Anaesthesiology and Cardiovascular Sciences, Sapienza Università di Roma, Rome, Italy; ^3^Laboratory Affiliated to Istituto Pasteur Italia - Fondazione Cenci Bolognetti, Rome, Italy; ^4^Laboratory of Cardiovascular Endocrinology, IRCCS San Raffaele Pisana, Rome, Italy; ^5^UOC, Clinical Pathology, San Giovanni Addolorata Hospital, Rome, Italy; ^6^Flow Cytometry Unit, IRCCS San Raffaele Pisana, Rome, Italy; ^7^Cardiology Unit, Department of Clinical and Molecular Medicine, Sant'Andrea Hospital, Sapienza University of Rome, Rome, Italy; ^8^Unit of Vascular Surgery, Department of Clinical and Molecular Medicine, Sant'Andrea Hospital, Sapienza University of Rome, Rome, Italy; ^9^Cell Stress and Survival Unit, Center of Autophagy, Recycling and Disease (CARD), Danish Cancer Society Research Center, Copenhagen, Denmark; ^10^Unit of Human Anatomy, Department of Anatomy, Histology, Forensic Medicine and Orthopaedics, Sapienza University of Rome, Rome, Italy; ^11^Department of Paediatric Haematology and Oncology, IRCCS Bambino Gesù Children's Hospital, Rome, Italy; ^12^Department of Biology, University of Rome Tor Vergata, Rome, Italy; ^13^Department of Human Sciences and Promotion of the Quality of Life, San Raffaele Roma Open University, Rome, Italy

**Keywords:** atherosclerosis, autophagy, ApoE-KO, regulatory T cells, plaques

## Abstract

Atherosclerosis is a progressive vascular disease representing the primary cause of morbidity and mortality in developed countries. Formerly, atherosclerosis was considered as a mere passive accumulation of lipids in blood vessels. However, it is now clear that atherosclerosis is a complex and multifactorial disease, in which the involvement of immune cells and inflammation play a key role. A variety of studies have shown that autophagy—a cellular catalytic mechanism able to remove injured cytoplasmic components in response to cellular stress—may be proatherogenic. So far, in this context, its role has been investigated in smooth muscle cells, macrophages, and endothelial cells, while the function of this catabolic protective process in lymphocyte functionality has been overlooked. The few studies carried out so far, however, suggested that autophagy modulation in lymphocyte subsets may be functionally related to plaque formation and development. Therefore, in this research, we aimed at better clarifying the role of lymphocyte subsets, mainly regulatory T cells (Tregs), in human atherosclerotic plaques and in animal models of atherosclerosis investigating the contribution of autophagy on immune cell homeostasis. Here, we investigate basal autophagy in a mouse model of atherosclerosis, apolipoprotein E (ApoE)-knockout (KO) mice, and we analyze the role of autophagy in driving Tregs polarization. We observed defective maturation of Tregs from ApoE-KO mice in response to tumor growth factor-β (TGFβ). TGFβ is a well-known autophagy inducer, and Tregs maturation defects in ApoE-KO mice seem to be related to autophagy impairment. In this work, we propose that autophagy underlies Tregs maturation, advocating that the study of this process in atherosclerosis may open new therapeutic strategies.

## Introduction

Atherosclerosis is a chronic inflammatory and multifactorial disease affecting the cardiovascular system and one of the leading causes of mortality in industrialized countries. Smoking, unhealthy diet, aging population, lack of physical activity, arterial hypertension, and diabetes promote atherosclerosis and possibly cardiovascular diseases such as myocardial infarction or stroke (1, 2). The early steps of atherosclerosis are characterized by the adhesion of blood leukocytes to the endothelial monolayer, the migration of the bound leukocytes into the intima, the maturation of monocytes into macrophages, and the subsequent enhanced uptake of lipids leading to their transformation into foam cells. Lesion progression involves the migration of smooth muscle cells (SMCs) from tunica media to intima. In advancing lesions, it has been reported that plaque macrophages and SMCs undergo cell death and contribute to the progression of the plaque (3–5). Extracellular lipids accumulate in the central region of the plaque, forming the lipid or necrotic core, thus promoting the progression of the lesion and, eventually, the plaque rupture, thrombus formation, and the subsequent myocardial infarction or stroke (6, 7).

Although a strong involvement of immune system response has been reported in the pathogenesis of atherosclerosis, the cellular and molecular mechanisms involved and the crosslink between immune cells during atherogenesis remain unclear (8, 9).

It is commonly known that preserving tolerance, while preventing autoimmunity and chronic inflammation, is decisive for slowing down the onset of atherosclerosis (10, 11). Regulatory T cells (Tregs) are the main CD4 T-cell subpopulation responsible for the maintenance of immunologic tolerance by suppressing T-cell responses, preventing autoimmunity, and suppressing inflammatory and proatherogenic immune response (12–14). T-cell differentiation into Tregs is driven by the lineage-determining transcription factor forkhead box P3 (FOXP3) (15). The tumor growth factor-β (TGFβ), a cytokine secreted by many cell types, including macrophages, promotes FOXP3 expression in naïve peripheral CD4+ T cells and converts them into FOXP3-expressing Tregs (16, 17). Recently, it has been reported that Tregs deletion of autophagy-related gene 7 (Atg7) or autophagy-related gene 5 (Atg5) alters immune homeostasis, establishing autophagy as a central and intrinsic regulator of Tregs (18). Autophagy, a self-protecting cellular catabolic process appointed to the recycling of biomolecules, is interestingly one of the molecular processes involved in the pathology of atherosclerosis (19, 20). So far, three forms of autophagy have been described: macroautophagy, microautophagy, and chaperone-mediated autophagy; hereafter, we refer to macroautophagy as autophagy (21, 22). In early atherosclerotic plaques, autophagy preserves normal cellular function to protect cells against oxidative injury, metabolic stress, and inflammation (23–25). Conversely, autophagy defects in macrophages, SMCs and endothelial cells, lead to cell senescence and apoptosis and promote atherosclerosis onset and progression (26).

Based on this evidence showing that autophagy is a relevant factor in the function and homeostasis of cells in the immune system and that chronic inflammation leads to the progression of atherosclerotic plaque, we hypothesized that an altered autophagic flux during Tregs differentiation might contribute to altering the inflammatory state of the lesions, promoting atherosclerotic plaque progression. Apolipoprotein E (ApoE)-knockout (KO) mice are the elective animal model to study atherosclerosis (27). ApoE is a component of lipoproteins (LPs), a biochemical assembly whose primary purpose is to transport lipids, fat-soluble vitamins and cholesterol into the lymph system and then into the blood. Mice lacking ApoE show a significant increase in total plasma cholesterol compared to wild-type animals and display atherosclerotic plaque development (28). In ApoE-KO mice, the progression of atherosclerotic disease is accelerated by treatment with aldosterone, a hormone which regulates blood pressure and promotes vascular function (29, 30). In this research, we characterize lymphocyte populations in human and murine atherosclerotic plaque, and we investigate the role of autophagy in Tregs differentiation.

## Results

### Characterization of Human Atherosclerotic Plaques: Tregs Accumulation in AP and APR

**Figure 1:**
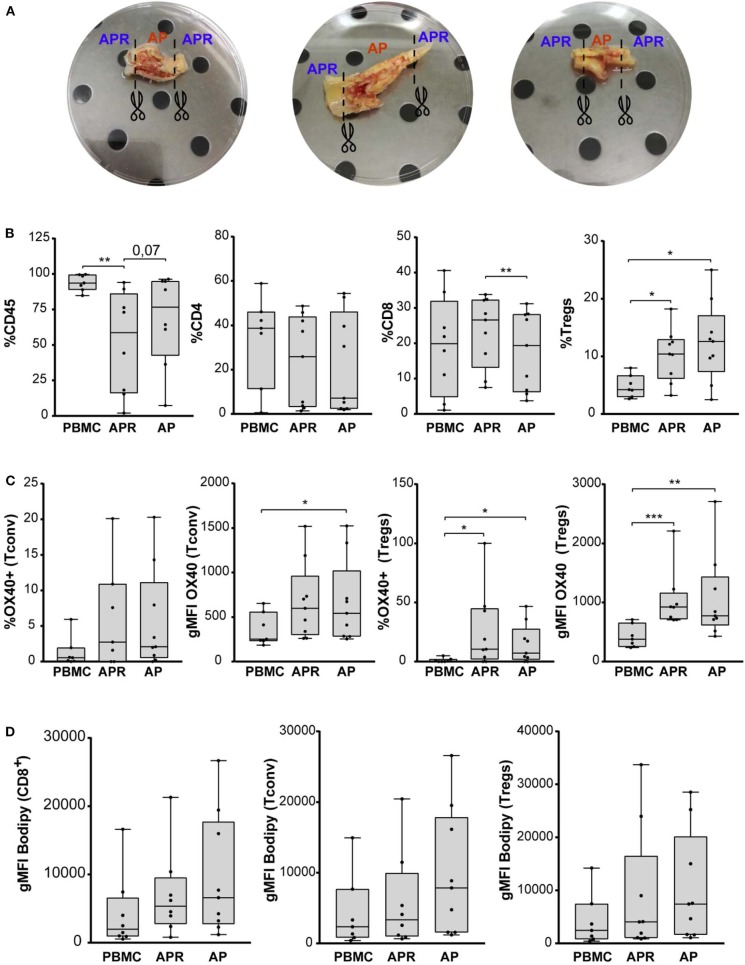
**(A)** Three representative carotid endarterectomy samples. The image shows sample division strategy: APR (adjacent atherosclerotic plaque region), AP (atherosclerotic plaque). **(B)** Frequency of CD45, CD4, CD8, and Tregs (in gated CD4+ T cells) in PBMC, APR, and AP from CEA patients (*n* = 9). **(C)** Percentage of OX40 in Tconv and Tregs and gMFI of OX40 gated on Tconv and Tregs. **(D)** gMFI of BODIPY in CD8, Tconv, and Tregs. In all figures, Tregs have been identified as FOXP3+ CD127^low^ within the CD14– CD8– viability dye– CD4+ CD25+ gate. *P < 0.05, **P < 0.01, ***P < 0.001, by paired *t*-test, two-tailed.

As a starting point, we characterized lymphocyte populations in human atherosclerotic plaques obtained by endarterectomy, a surgical procedure to remove the atheromatous plaque from carotids (31). Donor's characteristics are presented in Table 1 (section Materials and Methods). As shown in Figure 1A, in order to better characterize Tregs diversity, each carotid artery has been divided into an adjacent atherosclerotic plaque region (APR) and atherosclerotic plaque (AP), using peripheral blood mononuclear cells (PBMC) as a control of circulating immune cells. We performed a multiparameter flow cytometry analysis as described in Supplementary Table 1.

**Table 1:** Characteristics of all carotid endarterectomy (CEA) patients included in the study (*n* = 9).

		**Stenosis**		**Diseases**							
**Pt**	**Sex**	**Surgery side**	**Opposite surgery side**	**Hypertension**	**Diabetes**	**Mi**	**POA**	**Dyslipidemia**	**COPD**	**Smoke**	**Statins**
1	F	Left	80%	Yes	No	No	No	Yes	No	No	Yes
2	M	Left	90%	Yes	Yes	No	No	Yes	No	Yes	Yes
3	M	Right	80%	Yes	No	No	No	Yes	No	No	Yes
4	M	Right	90%	Yes	No	No	Yes	No	No	Yes	No
5	F	Right	70%	Yes	Yes	Yes	No	Yes	No	Yes	Yes
6	F	Right	80%	Yes	No	No	No	Yes	Yes	Yes	Yes
7	F	Right	70%	Yes	No	No	No	Yes	Yes	Yes	Yes
8	M	Right	70%	Yes	No	No	No	Yes	Yes	Yes	No
9	M	Left	80%	Yes	No	No	No	Yes	Yes	Yes	Yes

*The surgery side represents carotid occlusion (%); the opposite surgery side represents the condition of the opposite carotid artery. MI, Myocardial infarction; POA, Peripheral obliterant arteriopathy; COPD, Chronic obstructive pulmonary disease*.

We estimated the frequency (%) of leukocyte populations (CD45+), CD4+ T cells (CD4+CD8–), CD8+ T cells (CD8+CD4–), conventional T (Tconv) cells (CD127+/–FOXP3–/CD4+ T cells), and Tregs (CD127^low^FOXP3+/CD4+ T cells) by flow cytometry using the gating strategy shown in Supplementary Figure 1. We observed a significant decrease of CD45+ cells in APR compared to PBMC and a significant decrease of CD8+ T cells in AP compared to APR, while CD4+ cells were comparable among the experimental groups. Interestingly, the frequency of Tregs was significantly increased in APR and in AP, when compared to PBMC, suggesting a higher frequency of Tregs in the atherosclerotic plaque microenvironment (Figure 1B). We did not observe any differences in Tregs frequency between AP and APR, indicating that infiltration of Tregs takes place also in the adjoining plaque region.

Recent observation highlighted the pivotal role of the expression of OX40, a member of the TNFR/TNF superfamily, on activated CD4+ and CD8+ T cells (32), in supporting Tregs fitness in mouse models of homeostatic proliferation and colitis and in promoting the expansion of stable and suppressive Tregs in human cancers (33–35). Based on these findings, we investigated whether Tregs and Tconv in AP were characterized by higher levels of OX40 expression (Figure 1C). The geometric mean fluorescence intensity (gMFI) of Tconv-OX40+ was significantly increased in AP if compared to PBMC. Furthermore, we found a significant increment of the frequency and the gMFI of Tregs-OX40+ in AP and APR when compared to PBMC (Figure 1C). These results suggest that Tregs in the atherosclerotic plaque microenvironment expressed higher levels of OX40, possibly contributing to Tregs expansion.

It has been recently demonstrated that, besides inducing a huge frequency of Tregs, OX40 expression was associated with fatty acid accumulation and with the promotion of selective proliferation of lipid-laden Tregs (36). Based on this evidence, we stained the cells with BODIPY, a cell-membrane-permeable fluorophore specific for neutral lipid stores; however, we did not find any significant difference in the intracellular lipid content of CD8+ T cells, Tconv, and Tregs derived from both APR and AP when compared to PBMC (Figure 1D).

Overall, these results show that a significant fraction of Tregs are found in APR and even more in the AP site when compared to PBMC, suggesting Tregs-mediated modulation of inflammation at the atherosclerotic plaque and its associated region.

### Characterization of Atherosclerotic Plaques in ApoE-KO Mice Treated With Aldosterone

**Figure 2:**
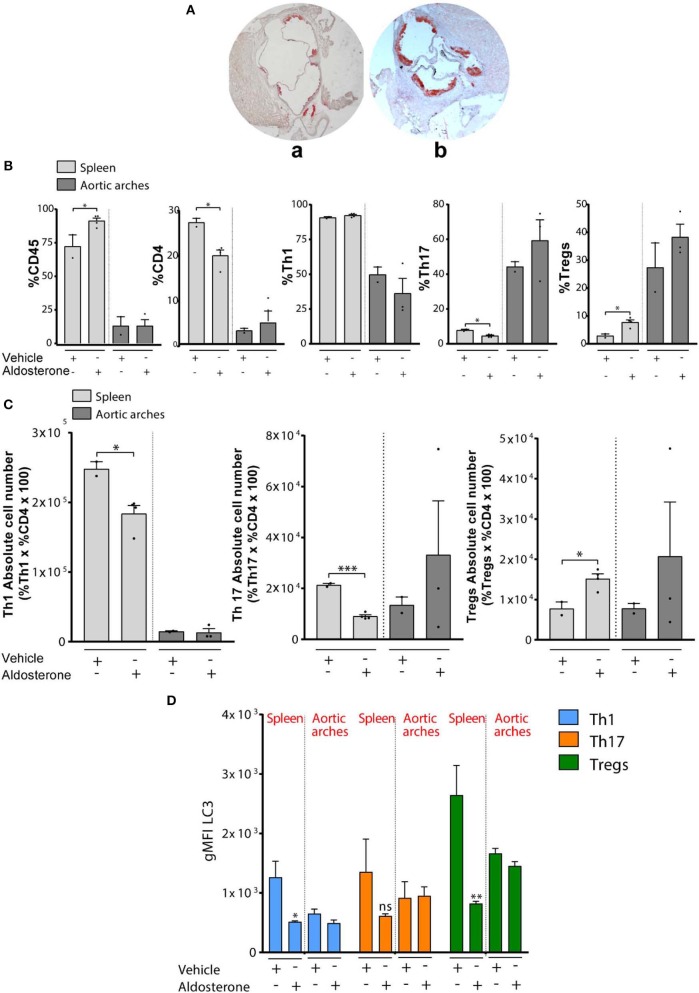
**(A)** Quantification of atherosclerotic plaque composition in vehicle- or aldosterone-treated ApoE-KO: Representative Red Oil O stained cross sections of aortic root in 8- to 10-week-old ApoE-KO mice treated with vehicle (a) or aldosterone (6 μg/mouse/day) (b) and fed an atherogenic diet for 4 weeks. **(B)** Percentage of CD45, CD4, CD8 Th1, Th17, and Tregs (in gated CD4+ T cells) in spleens and aortic arches from ApoE-KO mice treated or not with aldosterone (*n* = 8, four mice each group). **(C)** Absolute number of Th1, Th17, and Tregs calculated on the percentage in **(B)**. **(D)** gMFI of LC3 in Th1, Th17, and Tregs. In all figures, Th1, Th17, and Tregs have been identified as CXCR3+CCR6–CD4+, CCR6+CXCR3–CD4+, and FOXP3+CD4+, respectively, within the CD8– CD4+ gate. *P < 0.05, **P < 0.01, ***P < 0.001, by unpaired *t*-test, two-tailed.

To confirm our results *in vivo*, we took advantage of the ApoE-KO mouse, one of the most widely used models to study atherosclerosis (37). ApoE is a glycosylated protein, mainly bound to plasma lipoproteins but also expressed in hematopoietic stem and progenitor cells (HSPC) (37–39). In order to better resemble the human cardiovascular disease and to speed up plaque progression, we treated ApoE-KO mice with aldosterone at a dose of 6 μg/kg/day. Such concentration is able to raise serum aldosterone levels to a range which promotes atherosclerosis, without inducing hypertension (27, 30, 40). Aldosterone promotes rapid lipid accumulation in the aortic arch section of ApoE-KO mice, as shown in Figure 2A. In order to estimate the frequency of leukocytes (CD45+), CD4 T cells (CD4+CD3+CD45+), Th1 (CXCR3+CCR6–CD4+), Th17 (CCR6+CXCR3–CD4+), and Tregs (FOXP3+CD4+), we performed multiparameter flow cytometry (Supplementary Table 2) on splenocytes and aortic arches obtained from 8-week-old ApoE-KO mice treated with aldosterone or vehicle for 4 weeks (the gating strategy is shown in Supplementary Figure 2A).

We first observed a significant increase of CD45+ cells and a significant decrease of CD4+ T cells in splenocytes of ApoE-KO mice treated with aldosterone (Figure 2B). Gated on CD4+ T cells, we checked the frequency of Th1 cells (CD4+CXCR3+), Th17 cells (CD4+CCR6+), and Tregs (CD4+FOXP3+), and we found a significant decrease of Th17 and a significant increase of Tregs in ApoE-KO mouse spleens treated with aldosterone (Figure 2B). However, we did not observe significant differences of Th17 and Tregs percentages in aortic arches. The calculation of the absolute cell numbers (%Cells of interest × CD4 × 100) of different T-cell populations revealed a statistically significant decrease in Th1 cells and Th17 and a significant increase of Tregs in spleens of ApoE-KO mice treated with aldosterone (Figure 2C). In agreement with previous results, the absolute cell number in aortic arches does not show any significant difference. Overall, these data show that Tregs accumulate in the spleens of ApoE-KO mice under the atherosclerotic development condition (with aldosterone treatment), whereas no changes in the presence of Tregs were observed in ApoE-KO mouse atherosclerotic plaque.

In order to investigate the possible role played by autophagy in lymphocyte homeostasis and in T-cell polarization, we analyzed the levels of microtubule-associated proteins 1A/1B light chain 3B (LC3), as a marker of autophagy in splenocytes and aortic arches obtained from 8-week-old ApoE-KO mice in the presence or absence of aldosterone. In particular, we detected the phosphatidylethanolamine-conjugated form of LC3, named LC3-II, which is closely correlated with the number of autophagosomes and serves as a good indicator of autophagosome formation (41). We then calculated gMFI LC3-II by flow cytometric analysis, and we observed a significant decrease of LC3-II in Th1 and Tregs in ApoE-KO mouse spleens treated with aldosterone (Figure 2D); however, similar to what was observed before, we did not find any difference in aortic arches of ApoE-KO mice in the presence or absence of aldosterone. A decrease in LC3-II levels could be an indication of (i) impaired autophagy, (ii) enhanced autophagic flux, or (iii) decreased LC3 transcription. We performed additional experiments on isolated lymphocytes to investigate this aspect.

### Impaired Tregs Polarization in ApoE-KO Mice After TGFβ Stimulus

**Figure 3:**
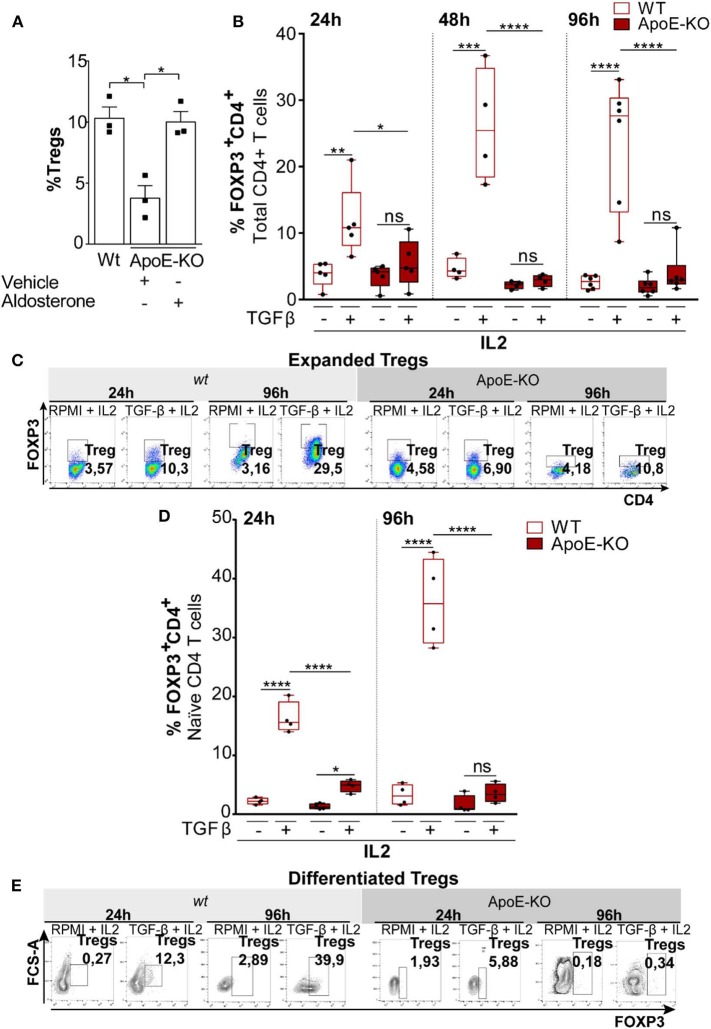
**(A)** Percentage of Tregs (FOXP3+CD4+) in splenocytes obtained from *wt* (*n* = 3), ApoE-KO (*n* = 3), and ApoE-KO + aldosterone (*n* = 3). **(B)** Expanded Tregs treated with TGFβ (2 ng/ml) and IL2 (100 U/ml) in *wt* (*n* = 5) and ApoE-KO mice (*n* = 5) started from CD4+ T cell. The histograms represent % of Tregs (CD4+FOXP3+) analyzed by flow cytometry. The experiments were conducted at different times, 24, 48, and 96 h, by culturing CD4+ T cells isolated from spleens of wild type (*wt*) mice and ApoE-KO mice, in the presence of IL2, treated or not with TGFβ and stimulated with plate-bound anti-CD3 and anti-CD28. **(C)** Representative dot plots of expanded Tregs from five independent experiments in *wt* and ApoE-KO mice. **(D)** Differentiated Tregs with TGFβ (2 ng/ml) and IL2 (100 U/ml) in *wt* (*n* = 4) and ApoE-KO mice (*n* = 4). The bar graph indicates % of Tregs (CD4+FOXP3+) at two different times, 24 and 96 h, by culturing naïve CD4+ T cells isolated from spleens of *wt* mice and ApoE-KO mice, in the presence of IL2, treated or not with TGFβ and stimulated with plate-bound anti-CD3 and anti-CD28. **(E)** Representative dot plots of differentiated Tregs from four independent experiments. *P < 0.05, **P < 0.01, ***P < 0.001, ****P < 0.0001, by two-way analysis of variance (ANOVA).

Based on our results and previous studies about Tregs differentiation during atherosclerotic plaque progression, we estimated the frequency of Tregs (FOXP3+CD4+ T cells) by flow cytometry in splenocytes derived from 8-week-old C57BL/6 wild-type (*wt*) and ApoE-KO mice treated or not with aldosterone. In line with previous literature (42), we observed a reduction of the percentage of Tregs in ApoE-KO mice compared with *wt* mice (Figure 3A). Surprisingly, we found that the frequency of Tregs (CD4+FOXP3+ gated on CD4+ T cells) in splenocytes was significantly increased in ApoE-KO mice when treated with aldosterone (Figure 3A). To clarify if the differences observed derive from aldosterone treatment itself or from the atherogenic-prone background, naïve CD4 T cells from *wt* mice were cultured in the presence of aldosterone for 96 h and with TGFβ to stimulate Tregs differentiation. However, as shown in Supplementary Figures 2B–**D**, no significant differences have been observed upon aldosterone treatment either in %CD4 T cells or in %Tregs, suggesting that the genetic background of ApoE-KO mice, rather than aldosterone treatment, is the determinant of the differential percentage of circulating Tregs. Although this is an interesting result, for subsequent *in vitro* experiments, we isolated cells from ApoE-KO mice that were not treated with aldosterone, in order to focus only on the genetic background. Our data, indeed, suggest that the aldosterone effect is presumably not directly interfering with immune cells but rather with the hormonal response in the animal that we exploited solely to speed up plaque formation (43). Hereby, we focused on the differences in Tregs frequencies between *wt* and ApoE-KO genetic background.

Tregs differentiation and expansion can occur extrathymically from naïve CD4+ T-cell precursors upon antigenic stimulation (44). Given the differences between *wt* and ApoE-KO mice in the frequency of Tregs, we first investigated the differences either in expanding Tregs starting from total CD4+ T cells or in the differentiating Tregs starting from naïve CD4+ T cells obtained from *wt* and ApoE-KO mice. Total CD4+ T cells (>5% FOXP3+), taken as a heterogeneous population derived from *wt* and ApoE-KO mouse spleens, were cultured for different times (24, 48, and 96 h) with interleukin-2 (IL2) in the presence or absence of TGFβ. Since CD4+ T cells include naïve CD4+ T cells, the precursors of Tregs, we could not determine the difference in proliferating Tregs or in induced Tregs. However, we observed a significant Tregs expansion in *wt* mice, already detected after 24 h of TGFβ treatment, reaching a higher extent after 96 h in culture (Figures 3B,C). In contrast, the treatment with TGFβ of ApoE-KO CD4+ T cells did not show any Tregs expansion. In line with this observation, the frequency of Tregs induced by TGFβ in *wt* was significantly much higher with respect to ApoE-KO cells. Calculation of Tregs absolute cell number (%Tregs × %CD4 × 100) shows how Tregs derived from *wt* animals were responsive to TGFβ stimulus differently from ApoE-KO cells (Supplementary Figure 3A). In order to quantify flow cytometry data, we also evaluated the geometric mean of FOXP3 which showed a significant reduction of intensity in ApoE-KO mice at 48 and 96 h, in agreement with the previous results (Supplementary Figure 4).

Considering that naïve CD4+ T cells are the precursors of both effector and memory T-cell subsets and that Tregs can be generated in the thymus or can differentiate from peripheral naïve CD4+ T cells (45), we stimulated Tregs polarization by culturing naïve CD4+ T cells obtained from *wt* and ApoE-KO mouse spleens (46). In particular, we cultured naïve CD4+ T cells in the presence of IL2 for 24 and 96 h, adding or not TGFβ and maintaining the same conditions used for CD4+ T cells. We found that TGFβ treatment did not induce ApoE-KO CD4+ Tregs differentiation, while *wt* cells respond to the treatment at both 24 and 96 h (Figures 3D,E). Thus, the frequency of Tregs induced by TGFβ in *wt* was significantly much higher with respect to ApoE-KO cells. To better appreciate the differences between *wt* and ApoE-KO, we calculated the Tregs absolute cell number. In accordance with the data previously shown, Tregs differentiation from *wt* mice cells was detectable after 24 h of TGFβ treatment, reaching the highest levels after 96 h. Although the absolute cell number of Tregs differentiation in ApoE-KO mice increased upon TGFβ treatment at both 24 and 96 h, the extent was much lower with respect to *wt* counterparts, thus suggesting a deficiency in TGFβ-induced maturation of ApoE-KO Tregs (Supplementary Figure 3B). In summary, our results show that Tregs differentiation in ApoE-KO is significantly impaired compared to *wt* counterparts.

### TGFβ Modulates Autophagy in Tregs

**Figure 4:**
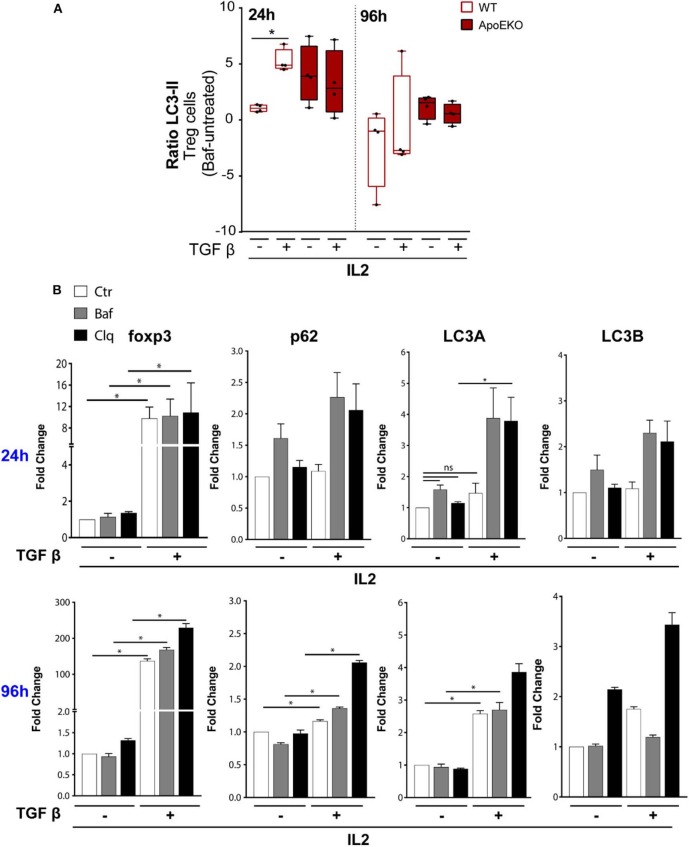
**(A)** Autophagic flux during Tregs differentiation in *wt* (*n* = 4) and ApoE-KO mice (*n* = 4). Tregs were induced by culturing naïve CD4+ T cells isolated from splenocytes, in the presence of IL2 (100 U/ml) treated or not with TGFβ (2 ng/ml) and stimulated with plate-bound anti-CD3 and anti-CD28. The histograms show flow cytometry analysis for 24 and 96 h with and without bafilomycin (represented as LC3-II ratio: Baf-untreated) during the last 4 h of stimulation. *P < 0.05, **P < 0.01, ***P < 0.001, by two-way analysis of variance (ANOVA). **(B)** Quantitative RTqPCR analysis of TGFβ-induced Tregs from naïve CD4+ T cells isolated from *wt* mice, in the presence of IL2 and stimulated with plate-bound anti-CD3 and anti-CD28 for 24 h (*n* = 3, 2 mice per experiment) and 96 h (*n* = 2, 8 mice per experiment). All the experiments were performed with and without bafilomycin (100 nM) or chloroquine (40 μM) during the last 4 h of differentiation. *P < 0.05, **P < 0.01, ***P < 0.001, by unpaired *t*-test, two-tailed.

Since autophagy is an important mechanism protecting Tregs lineage and survival integrity, we tested the hypothesis that autophagy might affect Tregs differentiation in ApoE-KO mice (18). In order to determine if autophagy was related to Tregs differentiation and to analyze its contribution in the defective Tregs differentiation observed in ApoE-KO mice, we first investigated the kinetics of autophagy in TGFβ-induced Tregs derived from naïve CD4+ T cells. Cells were cultured in Tregs-polarizing conditions (IL2 treatment with or without TGFβ) and analyzed at two time points: 24 and 96 h. The autophagy flux has been analyzed by flow cytometry comparing the LC3-II amount in the presence and absence of the lysosomal inhibitor bafilomycin (Baf), added 4 h before the end of the experiment (47). In order to evaluate the autophagic flux of CD4+ T cells, we calculated the LC3-II ratio between Baf-treated and untreated cells. On expanded Tregs gated on CD4, we did not observe any significant difference in the LC3-II ratio analyzed at two different time points: 24 and 96 h (data not shown). We therefore repeated the analysis during Tregs differentiation induced from naïve CD4+ T cells isolated from *wt* and ApoE-KO mice. We observed that the LC3-II ratio significantly increased in *wt* Tregs upon TGFβ treatment at 24 h, while no difference was observed in ApoE-KO Tregs (Figure 4A). These results indicate that TGFβ induces autophagy in *wt* but not in ApoE-KO Tregs. In particular, by blocking the autophagosome–lysosome fusion by Baf, 4 h before the end of the experiment, ApoE-KO mice did not show any significant increase in LC3-II ratio when challenged with TGFβ, suggesting a block of autophagic machinery during Tregs differentiation in ApoE-KO mice (Figure 4A).

We previously showed a reduction in LC3-II signal in ApoE-KO mouse spleen treated with aldosterone (Figure 2A). In order to exclude any commitment of aldosterone in LC3 lipidation in Tregs, we analyzed LC3-II-positive cells during Tregs differentiation by treating cells in the presence or absence of aldosterone at the same dose used for the *in vivo* experiment. After 96 h of Tregs differentiation, aldosterone did not interfere with LC3 lipidation (Supplementary Figure 5), suggesting that all the differences that we observed in the ApoE-KO mouse spleen, derived from the genetic background and not from the aldosterone treatment.

Due to the fact that Tregs derived from ApoE-KO mice seem to be autophagy impaired, we pharmacologically modulated autophagy in sorted *wt* naïve CD4+ T cells (CD4+CD62L+CD25–) under a TGFβ stimulus for the subsequent experiments. TGFβ has been reported to induce autophagy through the SMAD 2/3 pathway in different cell lines and by the increase in the mRNA levels of autophagic genes such as *BECLIN1, ATG5*, and *ATG7* (48, 49). Based on this knowledge, we performed RTqPCR at either 24 or 96 h by culturing naïve CD4+ T cells in IL2 in the presence or absence of TGFβ, in order to evaluate the mRNA levels of *Foxp3* and autophagic genes such as *p62, LC3A*, and *LC3B*. The obtained results showed an increase of mRNA levels of *Foxp3* after TGFβ and IL2 treatment at both 24 and 96 h (Figure 4B). As expected, the inhibition of autophagy by Baf or chloroquine, a lysosome acidification inhibitor, does not interfere with the transcriptional activation of *Foxp3* induced by TGFβ. We then investigated the mRNA levels of autophagic genes after 24 h of TGFβ and IL2 treatment; we did not observe any significant increase of *p62, LC3A*, and *LC3B*. Interestingly, at 24 h, the addition of Baf and chloroquine led to a significant increase of *LC3A* mRNA (Figure 4B, top), probably due to feedback mechanism of cells trying to overcome autophagy inhibition. The mRNA expression of *Foxp3*, already boosted by TGFβ at early time points, was remarkably increased at 96 h, as well as the mRNA expression of autophagy-related genes (*p62* and *LC3A*), confirming the transcriptional activation of autophagy by TGFβ (Figure 4B, bottom).

### FOXP3 Accumulates Upon Autophagy Inhibition

**Figure 5:**
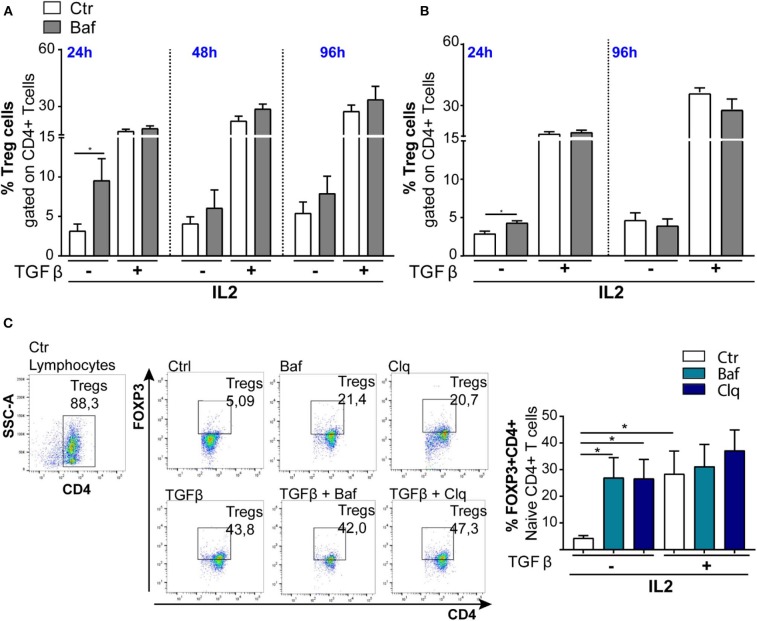
Induction of Tregs with TGFβ (2 ng/ml) in *wt* mice (*n* = 3): The bar graphs represent % of Tregs (CD4+FOXP3+) analyzed by flow cytometry. The experiments were conducted at different times, 24, 48, and 96 h, by culturing CD4+ T cells **(A)** and naïve CD4+ T cells **(B)** in the presence of IL2, treated or not with TGFβ and stimulated with plate-bound anti-CD3 and anti-CD28. **(C)** Representative dot plots of FOXP3+ induction from three animals. *P < 0.05, by unpaired *t*-test, two-tailed.

Autophagy is a crucial process controlling the turnover of proteins and organelles. In our experiments, we noticed that the percentage of Tregs (CD4+FOXP3+) was significantly increased after Baf treatment when added during the last 4 h of differentiation (Figures 5A,B) even if the same treatment did not affect the transcription of *Foxp3* gene. Therefore, we investigated whether FOXP3 levels were affected by autophagy. The main pathway through which FOXP3 is degraded relies on the ubiquitination on lysine-48 and its subsequent proteasome degradation. However, several evidence reported that the natural p300 inhibitor, garcinol, can induce FOXP3 degradation via a lysosome-dependent pathway (50, 51). In order to investigate more in depth the potential autophagy-mediated FOXP3 degradation, we cultured sorted *wt* naïve CD4+ T cells (CD4+CD62L+CD25–) obtained from mouse spleens in the presence or absence of TGFβ and IL2 for 96 h, and we treated them with Baf or chloroquine for the last 4 h, in order to block the last steps of the autophagic flux. By flow cytometry analysis, we observed that the treatment with autophagy inhibitors led to an increase of FOXP3 in unstimulated cells after 96 h in culture upon 4-h treatment with Baf or chloroquine (Figure 5C), while the differences were abolished after TGFβ treatment, presumably because, after 96 h of stimulation, the differentiation was complete, autophagy shut down, and *Foxp3* gene upregulated hundred-folds by the effect of the cytokine (as shown in Figure 4B). Although further experiments are needed to confirm the autophagy-mediated FOXP3 turnover, these data suggest that this transcription factor can be an autophagy target.

We additionally investigated this aspect in ApoE-KO mice, particularly by culturing mechanically sorted naïve CD4+ T cells in the presence or absence of Baf and TGFβ. However, we did not observe any FOXP3 accumulation with only Baf treatment (data not shown) probably due to both lack of Tregs differentiation upon TGFβ treatment and, as previously shown in Figure 4A, autophagy being blocked in ApoE-KO mice at both 24 and 96 h.

### Autophagy Affects Tregs Differentiation

**Figure 6:**
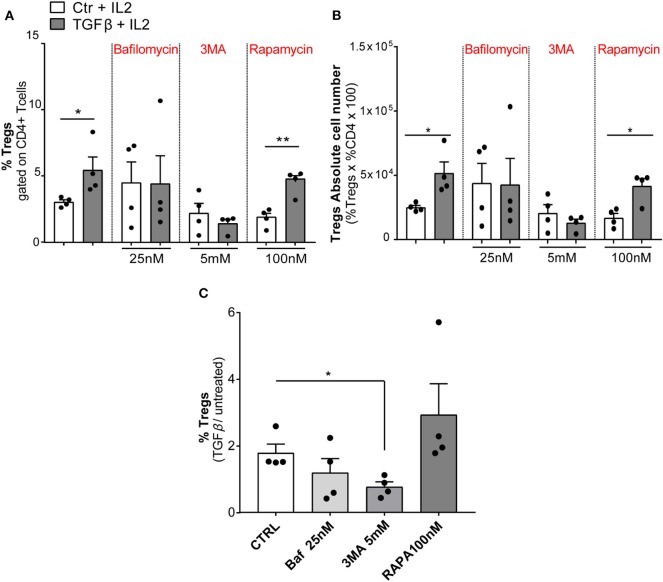
Modulation of autophagic flux during regulatory T-cell (Treg) differentiation. Tregs were induced by culturing naïve CD4+ T cells isolated from *wt* mice (*n* = 4, two mice per experiment) in the presence of IL2 (100 U/ml), treated or not with TGFβ (2 ng/ml) and stimulated with plate-bound anti-CD3 and anti-CD28 for 24 h. The histograms show the frequency of Tregs **(A)**, absolute number of Tregs **(B)**, and the result represented as normalization TGFβ/untreated **(C)**. The experiment was performed with autophagy modulators: bafilomycin (25 nM), 3-methyladenine (3MA, 5 mM), and rapamycin (100 nM) for 24 h. *P < 0.05, **P < 0.01, by unpaired *t*-test, two-tailed.

Finally, we modulated autophagy on sorted *wt* naïve CD4+ T cells (CD4+CD62L+CD25–) under a TGFβ stimulus in order to ascertain the involvement of autophagy in Tregs differentiation. We used for 24 h Baf or 3-methyladenine (3MA) (a PI3 kinase inhibitor) to inhibit autophagy or, alternatively, rapamycin (RAPA) [the inhibitor of the main physiological autophagy suppressor mammalian target of RAPA (mTOR)] to boost autophagic flux (52, 53). As shown in Figures 6A,B, both Baf and 3MA were able to inhibit differentiation of Tregs induced by TGFβ, while autophagy activation by RAPA has no effects on Tregs differentiation, presumably because autophagy was already activated by TGFβ. We obtained the same results by quantifying the percentage of FOXP3+ cells (Figure 6A) or the absolute Tregs cell number (Figure 6B). Finally, to better appreciate the results, we normalized the percentage of FOXP3 in TGFβ-treated or untreated samples, and we observed that autophagy inhibition by 3MA led to a significant decrease of Tregs differentiation (Figure 6C). Altogether, these results suggest that the modulation of autophagy impacts Tregs differentiation.

## Discussion

For many years, the therapy for the treatment of atherosclerosis has focused on lipid reduction methods. However, increasing evidence shows a strong involvement of the immune system in its pathogenesis and the idea of atherosclerosis as an autoimmune disease arose, paving the way toward new therapeutic approaches (54). A correlation between atherosclerosis and inflammation has been recognized in many studies, implying that immune cells and inflammatory signals are essential contributors to cardiovascular diseases (10, 55). In this scenario, it has been reported that Tregs exert atheroprotective properties by secretion of anti-inflammatory cytokines and suppression of T-cell proliferation (56). Coherently, atherosclerosis-prone mice harbor systemically less Tregs and a general imbalance of immune cells, potentially leading to atherogenesis (56). To add a further layer of complexity, it has been revealed that autophagy, the catabolic process responsible for the recycling of cellular components, when activated in immune cells within the atherosclerotic plaque preserves normal cellular function and protects plaque cells against oxidative injury, metabolic stress, and inflammation (26). Although the role of immune cells in regulating atherosclerosis onset and progression has been confirmed by many scientists, the contribution of autophagy in the maintenance of cellular homeostasis within atherosclerotic plaques is still neglected. With this research, we aimed at characterizing lymphocyte populations in human and murine plaques and addressing the effects of autophagy on Tregs differentiation in the context of atherosclerosis.

We started our study by characterizing human and mouse atherosclerotic plaques from an immunological point of view. The relative content of different lymphocyte subpopulations in the plaques, especially the frequency of the immunosuppressive Tregs, is crucial to understanding how the atherosclerotic disease progresses. Moreover, the establishment of immuno-profiles is of major importance in clinical practice as they could be used as predicting factor for cardiovascular atherosclerosis risk and therapy efficiency (57). Our initial characterization indicates that plaques from both human and animal models display more infiltrating Tregs. Notably, since the structural integrity of the artery wall is compromised also in the surrounding area of atherosclerotic plaques, we analyzed the regions adjacent to the lesion, and also in this case, we observed high frequencies of Tregs. In order to obtain fresh murine atherosclerotic plaques for our studies, we took advantage of ApoE-KO mice treated with aldosterone—so far, the elective mouse model for this disease (58). Our aim, using this experimental model of atherosclerosis, was merely to obtain samples to analyze from an immunologic point of view, and therefore, we did not compare our results with *wt* non-atherosclerosis vessels, from which we could not extract any infiltrating cells to relate. Remarkably, *wt* mice do not develop atherosclerosis either during aging or under aldosterone treatment. Our research did not aim to dissect the etiological mechanism giving rise to the plaque or the pathologies (e.g., hypertension) leading to atherosclerosis.

In line with other studies, our results corroborate evidence that the frequency of Tregs is decreased in splenocytes of ApoE-KO mice, suggesting that the development of atherosclerotic plaques in the mouse could be related to depletion of a peripheral Tregs pool (42). Surprisingly, we also revealed that ApoE-KO mice treated with aldosterone display instead an increase of peripheral Tregs, presumably due to their recruitment at the plaque region to suppress the inflammation promoted by aldosterone. It is well-known, indeed, that this hormone is associated with vascular infiltration of immune cells, reactive oxidative stress, and pro-inflammatory cytokine production (59). Here, we also demonstrate that, differently from *wt*, ApoE-KO mouse-derived CD4+ T cells are unable to differentiate in Tregs when stimulated with IL2 and TGFβ. This result clearly indicates that ApoE-KO mice show a defect in Tregs maturation that could, potentially, contribute to atherosclerosis progression.

In order to characterize mechanisms responsible for the defects of Tregs maturation in atherosclerotic plaque, we directed our studies toward the investigation of autophagy, due to its crucial role in sustaining the survival of immune cells at the level of atherosclerotic lesions. Although it is well-established that autophagy is essential for CD4+ T-cell proliferation, survival, cytokine production, and homeostasis in response to T-cell receptor activation (18, 60), the role of this catabolic process in Tregs maturation has been poorly dissected. Although far from being exhaustive, our experiments point toward a pivotal role of autophagy in Tregs differentiation that could inspire other researchers in this direction. We here show that TGFβ treatment in the presence of IL2, while inducing Tregs differentiation through FOXP3 transcription, triggers autophagy by inducing LC3 lipidation. At a later time point of stimulation, TGFβ is also responsible for the induction of the transcription of autophagic genes such as *p62* and *LC3A*. In order to functionally correlate autophagy induction with Tregs differentiation, we incubated naïve CD4+ T cells with autophagy pharmacological inhibitors (Baf or 3MA) for 24 h together with the differentiation mixture (IL2+TGFβ), and we observed an inhibition of the TGFβ-induced differentiation though autophagy induction, *per se*, with RAPA was not able to induce Tregs maturation. Overall, these data indicate that autophagy contributes to TGFβ-induced differentiation, but it is not the only driving force. In our experiments, we also noticed that short treatments with autophagic inhibitors lead to an accumulation of FOXP3, the main transcription factor driving Tregs differentiation, indicating it as a possible autophagy target and linking Tregs differentiation to autophagy.

Finally, and even more interestingly, we found that ApoE-KO mouse-derived CD4+ T cells that are unable to differentiate are also unable to activate autophagy upon TGFβ treatment. In this regard, we specify that lymphocytes derived from *wt* and ApoE-KO mice are phenotypically the same (ApoE expression in Tregs is virtually absent); however, they respond differently probably because of defects accumulated during the development in the mouse which persist even after we grow the cells in culture under controlled conditions. Dyslipidemia could, for instance, account for these lymphocyte defects, but this aspect was not investigated here. Moreover, the mechanism(s) leading to autophagy impairment in ApoE-KO mice is still unknown. However, with the data here provided, we can speculate that autophagy impairment (similarly to what is seen in other lymphocyte populations), by inhibiting Tregs maturation in ApoE-KO mice, enhances the inflammatory process at the level of atherosclerotic plaques.

In addition, seeking to analyze the autophagic flux in Tregs of human plaques, we checked the levels of phospho-S6 ribosomal protein kinase (p-S6)—a marker of mTOR pathway activation and autophagy inhibition—in Tregs cells derived from human plaques (61, 62). Although we observed an increase of p-S6 in a limited number of samples (data not shown), our knowledge regarding autophagy in Tregs of human plaques is still, unfortunately, not exhaustive.

Taken together, our results indicate that autophagy is a necessary process for Tregs differentiation and that the defective Tregs maturation in ApoE-KO mice could be connected to autophagy blockade. The present work underlines the complexity of the autophagy–Tregs–atherosclerosis axis and enlightens some important connections between immune cells and this catabolic process that could be exploited during the development of new prognostic and therapeutic strategies, which could be useful in rehabilitation programs for atherosclerotic patients. In conclusion, we suggest that, by enforcing the functional integrity of Tregs, the manipulation of autophagy may be exploited as a useful tool for the development of a novel therapeutic approach aimed at increasing functional Tregs in atherosclerosis therapy (18).

## Materials and Methods

### Patients

Peripheral blood (PB), adjacent APR, and AP samples were obtained from nine patients undergoing carotid endarterectomy (CEA) (63) affected by different pathologies representing atherosclerosis risk factors. Patient's characteristics are shown in Table 1, displaying the percentage of occlusion (surgery-side) and associated pathologies.

PBMC were isolated from the PB of CEA patients by density gradient centrifugation with Lympholyte (Cedarlane Laboratories USA, cat. #CL5020) and collected in complete RPMI Dutch-modified medium containing 10% FBS (Gibco), 2 mM l-glutamine (Sigma-Aldrich), penicillin/streptomycin (Gibco), non-essential amino acids, sodium pyruvate (EuroClone), and 50 μM 2-mercaptoethanol (Sigma-Aldrich).

Mononuclear cells were extracted from APR and AP. Fragments of APR and AP were digested with Collagenase IV (C5138 Sigma) (2 mg/ml) + DNase I (0.1 mg/ml) in RPMI for 1–2 h at 37°C. Single-cell suspensions were obtained by disrupting the fragments with a syringe plunger over a cell strainer and pelleted through a 40% isotonic Percoll solution. Human studies were performed in accordance with the General Data Protection and Regulation (GDPR) and ethical guidelines of the 1975 Declaration of Helsinki and were approved by the Institutional Ethical Committee of the University of Rome “La Sapienza” (authorization: RIF. 3720_2015/23.07.2015 Prot. 2372/2015).

### Mice

ApoE-KO mice were kindly provided by the Laboratory of Cardiovascular Endocrinology, IRCCS San Raffaele (Rome, Italy), maintained at Animal Facility Castel Romano (Rome) under protocols approved by the Italian Ministry of Health (493/2016-PR).

In 9-week-old male mice deficient for ApoE (ApoE-KO), osmotic minipump (Alzet model 1004) containing vehicle (ethanol/saline) or aldosterone (6 μg/mouse/day) was subcutaneously placed, and mice were fed with a proatherogenic HF diet (Harlan Teklad TD.88137) for 4 weeks (43). This dose of aldosterone was chosen on the basis of published studies demonstrating resultant serum aldosterone levels in physiologically relevant range, no change in blood pressure, and reproducible increases in atherosclerosis (30). At the time of sacrifice, animals were perfused with phosphate-buffered saline (PBS), and aortic valves were embedded in an optimal cutting compound (OCT) and collected at −80°C.

Cryosections of embedded aortic roots at the site where all three aortic valve leaflets could be visualized were taken at 10-μm intervals. Sequential sections were stained with Oil-Red O (ORO) in the aortic root at the level of the aortic valve.

To study the autophagy process, 8- to 12-weeks-old male C57BL/6J mice (Charles River Laboratories) housed at the animal facility of the Department of Anatomy, Histology, Forensic Medicine and Orthopaedics (DAHFMO), Unit of Histology and Medical Embryology (Sapienza University of Rome), were used. C57BL/6J mice were killed by CO_2_ euthanasia.

Eight- to ten-weeks-old male C57BL/6N mice (Taconic), housed at the animal facility at the Danish Cancer Society Research Center (Copenhagen, Denmark), were used to study autophagy modulation (e.g., Baf, 3MA, and RAPA treatments) and FOXP3 accumulation (RTqPCR and cytofluorometry analyses).

### Splenocyte Isolation and Aortic Arch Dissection From Murine Spleens

Characterization of ApoE-KO mouse spleens was performed in collaboration with the Laboratory of Cardiovascular Endocrinology, IRCCS San Raffaele (Rome, Italy). Splenocytes were obtained by spleen mechanical rupture with a syringe plunger followed by incubation with ACK (Lonza, Alpharetta, GA, USA, cat. no. 10-548E) for 5 min at room temperature (RT), and after washing, cells were filtered on a 70-μm cell strainer. The aortic arches were separated from the apical portion of the heart and were digested with collagenase IV (C5138 Sigma) (2 mg/ml) + DNase I (0.1 mg/ml) in RPMI 1640 for 1 h at 37°C.

### *In vitro* Analysis of Tregs Differentiation

Splenocytes were obtained by spleen mechanical rupture with a syringe plunger followed by incubation with sterile ddH_2_O for 10 s, blocking and washing with PBS 10×. CD4+ T cells were purified from splenocytes by using a CD4+ T-cell isolation kit (130-104-454 Miltenyi Biotec) and naïve CD4 T cells purified from splenocytes by using a naïve CD4+ T-cell isolation kit (130-104-453 Miltenyi Biotec) according to the manufacturer's instructions. Naïve CD4+ T cells and CD4+ T cells purified were cultured on 3 μg/ml of anti-CD3 (145-2C11) precoated wells (96-u bottom multiwell) at 37°C, at different time points (24, 48, and 96 h). Cells were plated in RPMI 1640 (R0883 Sigma) supplemented with 10% FBS, 2 mM l-glutamine, 100 U/ml penicillin, 100 μg/ml streptomycin, 10 mM HEPES, 50 μM 2-mercaptoethanol, 1 mM pyruvate, and 15 μg/ml gentamicin, in the presence of 2 μg/ml soluble anti-mouse CD28 (37.51 monoclonal antibody) and of human recombinant IL2 (354043 BD Bioscience) at 100 U/ml in the presence or absence of 2 ng/ml mouse recombinant TGFβ (14-8342 Affymetrix eBioscience). In some sets of experiments, cell cultures were treated with modulators of autophagy: Baf A1 (100 nM) (B1793 Sigma), chloroquine (40 μM) (C6626 Sigma), 3-methyladenine (5 mM) (M9281 Sigma), RAPA (100 nM) (R0395 Sigma). Aldosterone (10^−8^ M) treatment was used, maintaining the same conditions of the *in vitro* experiment (64).

### Flow Cytometry

Cells obtained from human atherosclerotic plaques and ApoE-KO mice were analyzed following the complete panel of antibodies (Tables S1, S2). Surface staining was performed by incubating the cells with selected antibodies at 4°C for 30 min in PBS. Intracellular staining of cytokines and transcription factors was performed with Foxp3 Transcription Factor Staining Buffer (eBioscience) in accordance with the manufacturer's instructions. Dead cells were excluded using Fixable Viability Dye eFluor® 780 (eBioscience). Human samples were acquired on the BD LSRFortessa cell analyzer (BD Biosciences), while mouse samples were run on the BD LSRFortessa X20 (BD Bioscience).

Regarding *in vitro* experiments, intracellular FOXP3 and LC3-II staining was performed using PFA 4% and 90–100% Met-OH for fixation and permeabilization, according to Cell Signaling protocol. Dead cells were excluded by staining with NIR (Live Dead Fixable Near-IR Dead Cell stain kit; Invitrogen) according to the manufacturer's instructions. Samples were run on a Cyan cytometer (Beckman Coulter).

Autophagic flux was measured by FACS analysis using antibody against LC3-II-PE (Cell Signaling clone D-11), in the presence or absence of the modulators of autophagy as described above.

Samples were analyzed with FlowJo software, version 10.5.3.

### Cell Sorting and RTqPCR

Naïve CD4+ T cells were stained with the antibodies CD4 eFluor-450, CD25 APC, and CD62L PECy7 and were sorted by using FACS Melody (BD).

Total RNA was extracted using the NucleoSpin RNA kit (740955.250 Macherey-Nagel). RNA concentration and purity were measured by NanoDrop (NanoDrop™ 2000/2000c spectrophotometers). Quantitative PCR was performed with 2 μl of cDNA, 0.4 μl of each primer (10 μmol/μl), and 10 μl of PowerUp SYBR Green qPCR Master Mix (Thermo Fisher Scientific) and analyzed with the QuantStudio software (Applied Biosystems).

The cycle threshold values were used to calculate the normalized expression of FOXP3, LC3A, LC3B, and p62 against β-actin.

The sequences of primer pairs are listed below:

mFOXP3f: AAT AGT TCC TTC CCA GAG TTC

mFOXP3r: GGT AGA TTT CAT TGA GTG TCC

mLC3Af: TTG GTC AAG ATC ATC CGG C

mLC3Ar: GCT CAC CAT GCT GTG CTG G

mLC3Bf: CCC ACC AAG ATC CCA GTG AT

mLC3Br: CCA GGA ACT TGG TCT TGT CCA

mSqstm1f: AAT GTG ATC TGT GAT GGT TG

mSqstm1r: GAG AGA AGC TAT CAG AGA GG

mActinf: CAC ACC CGC CAC CAG TTC GC

mActinr: TTG CAC ATG CCG GAG CCG TT.

### Statistical Analysis

Statistical analysis was performed using the version 7.0 Prism software (GraphPad). A two-tailed paired Student's *t*-test was used to analyze human data to compare PBMC, APR, and AP in the same sample. The two-tailed unpaired Student's *t*-test was applied to compare data from Th1, Th17, and Tregs in ApoE-KO vs. aldosterone. Two-way analysis of variance (ANOVA) was performed for *in vitro* analysis in which we compared *wt* vs. ApoE-KO mice upon TGFβ treatment. The two-tailed unpaired Student's *t*-test was applied to compare treatment with autophagy modulators when only one group was analyzed (*wt*). One-way ANOVA was performed for the *in vitro* experiment to analyze autophagic flux upon aldosterone treatment during Tregs differentiation. Every *in vitro* assay was performed in duplicate or triplicate, when possible. The number of repetitions is indicated in the figure legend for all experiments. In all graphs, bars show means ± SEM. In all tests, *P* < 0.05 was considered statistically significant.

## Data Availability Statement

All datasets generated for this study are included in the article/Supplementary Material.

## Ethics Statement

The studies involving human participants were reviewed and approved by Azienda Ospedaliera S. Andrea, Sapienza University of Rome—Rif. 3720_2015/23.07.2015. The patients/participants provided their written informed consent to participate in this study. The animal study was reviewed and approved by IRCCS San Raffaele (Rome, Italy), maintained at Animal Facility Castel Romano (Rome) under protocols approved by the Italian Ministry of Health (493/2016-PR).

## Author Contributions

AF, SP, FC, CG, and DS conceived the study. SM designed and performed the majority of the experiments and analyzed the data. FP contributed to the design of the multiparameter flow cytometry analysis. IP contributed to the experiments on human plaques. SM wrote the manuscript supported by SP and CG, and supervised by AF, MC, DD, and FC. FC and DD have hosted SM at the Danish Cancer Society Research Center (Denmark, DK) and contributed to the design of the experiments on autophagy. DD has supported SM to investigate autophagy and supervised the experiments regarding autophagy investigation. LV contributed to the design of the multiparameter flow cytometry in the murine model. MC, VM, CM, and AA provided the ApoE-KO mouse models and contributed to the experimental plan on the murine model. CS and MT provided the human atherosclerotic plaque and clinical characteristics of the patients. The manuscript was reviewed, revised, and edited by all authors.

### Conflict of Interest

The authors declare that the research was conducted in the absence of any commercial or financial relationships that could be construed as a potential conflict of interest.

## Supplementary Material

Supplementary Figure 1 Gating strategy for Tregs identification in PBMC, APR, AP by flow cytometry. Flow cytometry analysis was performed on lymphocytes extracted from **(A)** human peripheral blood mononuclear cells (PBMC), **(B)** adjacent Atherosclerotic Plaque Region (APR), **(C)** Atherosclerotic Plaque (AP). In gated CD4+ T cells, Tregs and Tconv were selected as FOXP3+ CD127^low^ within the CD14– CD8– viability dye- CD4+ CD25+ gate.

Supplementary Figure 2 **(A)** Gating strategy for Tregs identification in spleens and aortic arches obtained from ApoE-KO ± aldosterone (6 μg/mouse/day). In gated CD4+ T cells, Tregs, Th1 and Th17 were selected as CXCR3+CCR6-CD4+, CCR6+CXCR3–CD4+, FOXP3+CD4+, respectively, within the CD8– CD4+ gate. **(B)** Gating strategy for Tregs differentiation by culturing naïve CD4+ T cells isolated from *wt* mice (*n* = 4) and stimulated with plate-bound anti-CD3 and anti-CD28 for 96 h in presence of IL2 (100 U/ml), treated or not with TGFβ (2 ng/ml). In some set of experiment aldosterone [10^−9^ M] was added for 96 h. The histograms show the frequency of CD4+ T cells **(C)**, the frequency of Tregs represented as FOXP3+CD4+ cells (**D** left) and as TGFβ/Ctr fold change (**D** right).

Supplementary Figure 3 Absolute cell number of Tregs, calculated on percentage of expanded Tregs at different times (24, 48, and 96 h) **(A)** and differentiated Tregs at two timepoints (24 and 96 h) **(B)**, starting from CD4+ T cells of *wt* and ApoE-KO mice, in presence of IL2 (100 U/ml), treated or not with TGFβ (2 ng/ml) and stimulated with plate-bound anti-CD3 and anti-CD28.

Supplementary Figure 4 The bar graphs represent gMFI FOXP3 in Tregs analyzed by flow cytometry. The experiments were conducted at different times: 24, 48, and 96 h, by culturing CD4+ T cells in presence of IL2 (100 U/ml), treated or not with TGFβ (2 ng/ml) and stimulated with plate-bound anti-CD3 and anti-CD28.

Supplementary Figure 5 Autophagic flux during Tregs differentiation under aldosterone treatment in *wt* (*n* = 4) mice for 96 h. Tregs were induced by culturing naïve CD4+ T cells isolated from splenocytes, in presence of IL2 (100 U/ml) treated or not with TGFβ (2 ng/ml) and stimulated with plate-bound anti-CD3 and anti-CD28. In some set of experiments aldosterone was added for 96 h. The histogram shows the frequency of Tregs-LC3-II+ (FOXP3+LC3-II+) cells gated on CD4+ T cells during 96 h of aldosterone [10^−9^ M] treatment. To analyze autophagic flux in some sets of experiments bafilomycin was added for during the last 4 h of stimulation. One-way Analysis of Variance (ANOVA) was performed without showing any significant differences.

Supplementary Table 1 The multiparameter flow cytometry analysis was used to characterize human atherosclerotic plaque.

Supplementary Table 2 The multiparameter flow cytometry analysis was used to characterize mice spleens and aortic arches.
