# Supplementary figures and images for: Altered Tregs Differentiation and Impaired Autophagy Correlate to Atherosclerotic Disease
Source: Front Immunol. 2020 Mar 13;11:350. doi: 10.3389/fimmu.2020.00350 (PMC7082762; doi:10.3389/fimmu.2020.00350)

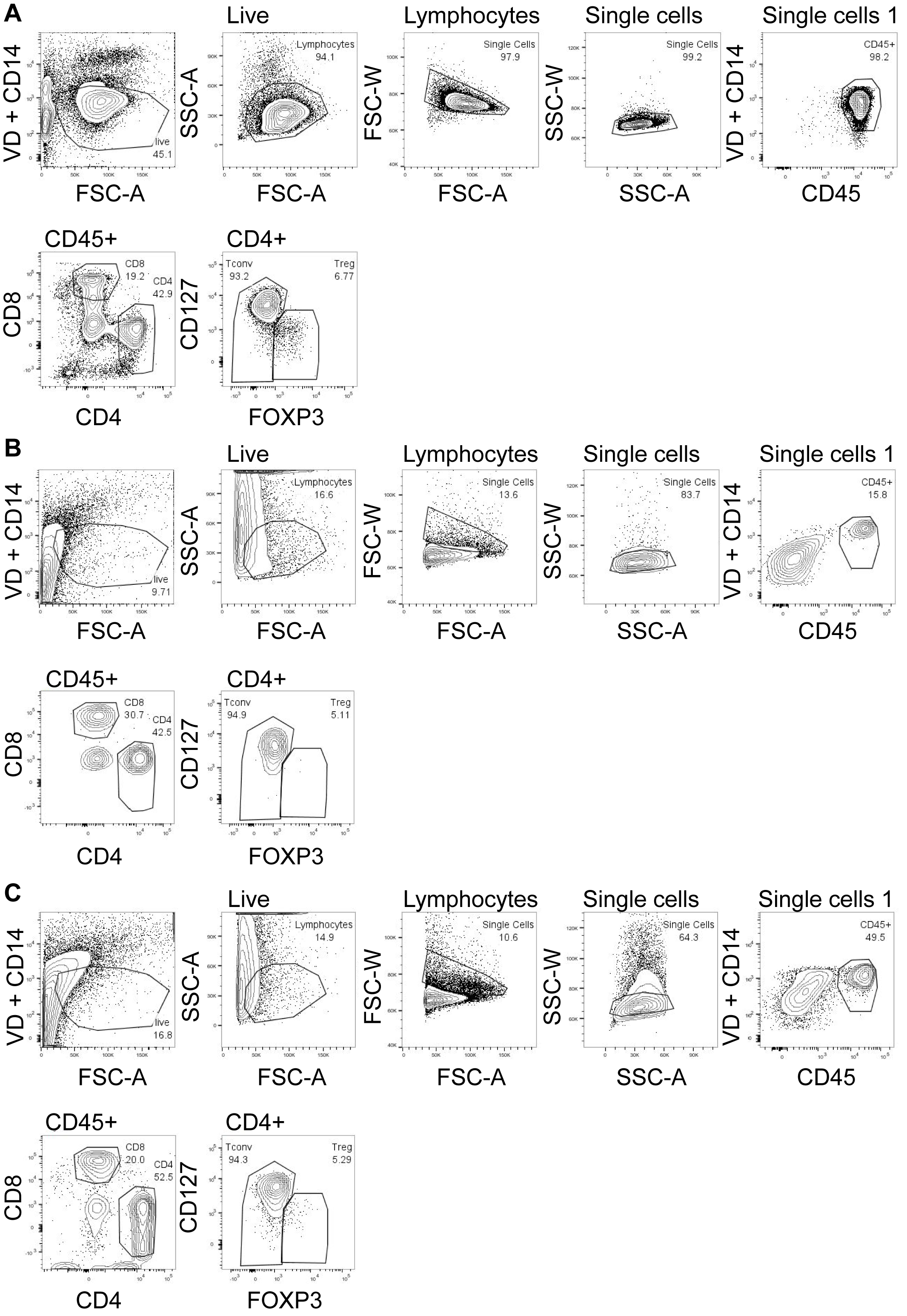

Supplement: Supplementary Figure 1 — Gating strategy for Tregs identification in PBMC, APR, AP by flow cytometry. Flow cytometry analysis was performed on lymphocytes extracted from (A) human peripheral blood mononuclear cells (PBMC), (B) adjacent Atherosclerotic Plaque Region (APR), (C) Atherosclerotic Plaque (AP). In gated CD4+ T cells, Tregs and Tconv were selected as FOXP3+ CD127low within the CD14– CD8– viability dye- CD4+ CD25+ gate. [file Image_1.TIF]

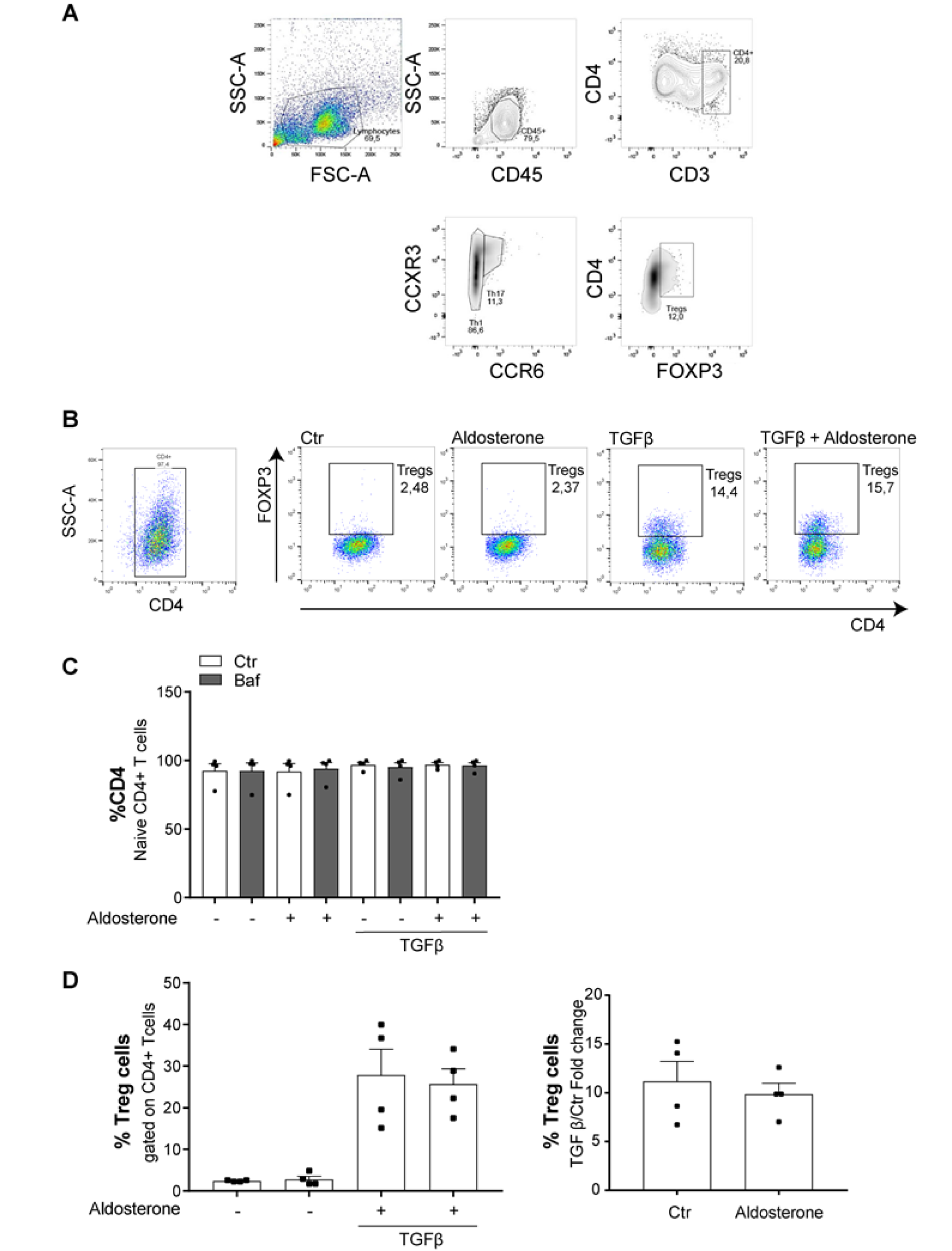

Supplement: Supplementary Figure 2 — (A) Gating strategy for Tregs identification in spleens and aortic arches obtained from ApoE-KO ± aldosterone (6 μg/mouse/day). In gated CD4+ T cells, Tregs, Th1 and Th17 were selected as CXCR3+CCR6-CD4+, CCR6+CXCR3–CD4+, FOXP3+CD4+, respectively, within the CD8– CD4+ gate. (B) Gating strategy for Tregs differentiation by culturing naïve CD4+ T cells isolated from wt mice (n = 4) and stimulated with plate-bound anti-CD3 and anti-CD28 for 96 h in presence of IL2 (100 U/ml), treated or not with TGFβ (2 ng/ml). In some set of experiment aldosterone [10−9 M] was added for 96 h. The histograms show the frequency of CD4+ T cells (C), the frequency of Tregs represented as FOXP3+CD4+ cells (D left) and as TGFβ/Ctr fold change (D right). [file Image_2.TIF]

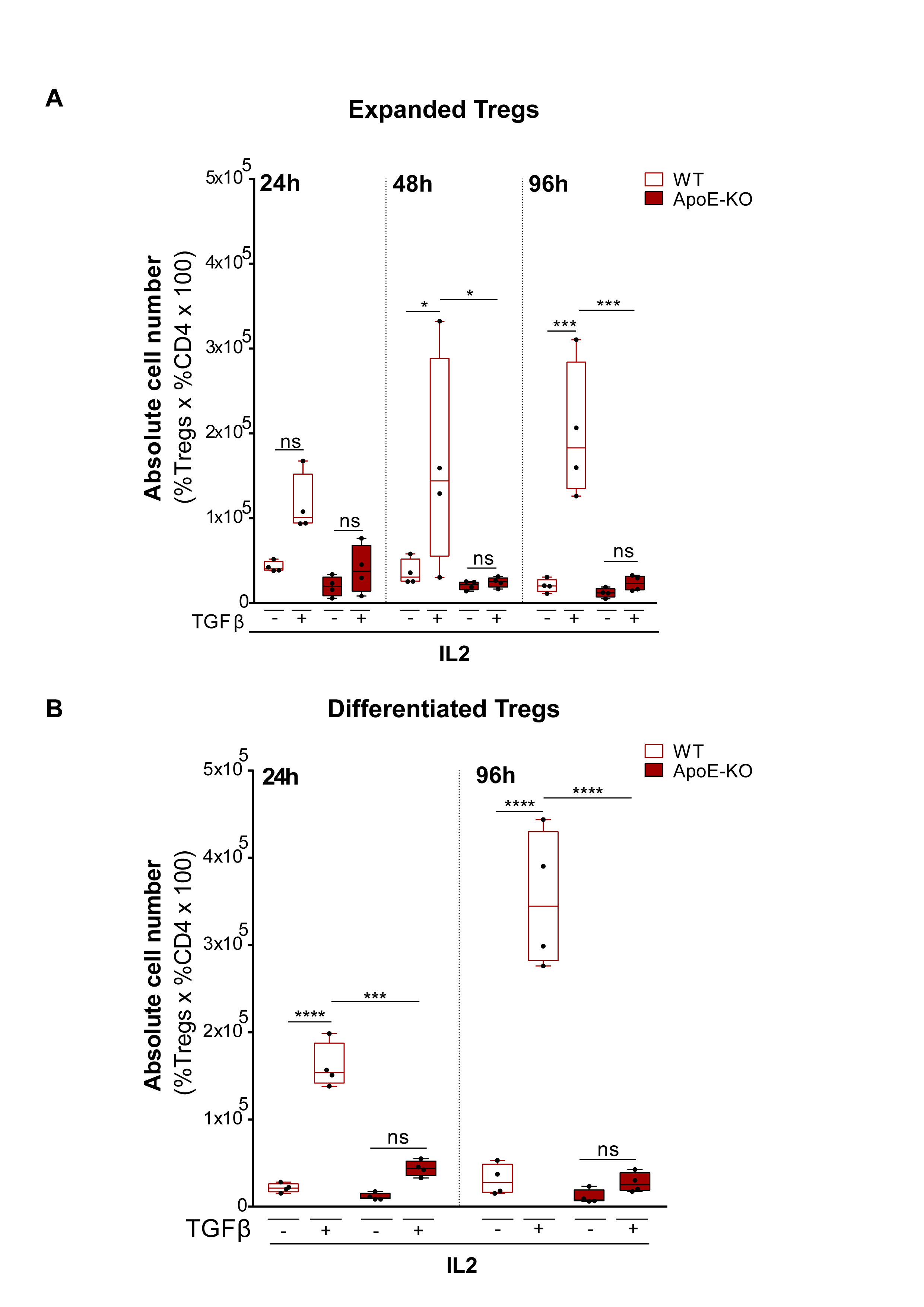

Supplement: Supplementary Figure 3 — Absolute cell number of Tregs, calculated on percentage of expanded Tregs at different times (24, 48, and 96 h) (A) and differentiated Tregs at two timepoints (24 and 96 h) (B), starting from CD4+ T cells of wt and ApoE-KO mice, in presence of IL2 (100 U/ml), treated or not with TGFβ (2 ng/ml) and stimulated with plate-bound anti-CD3 and anti-CD28. [file Image_3.TIF]

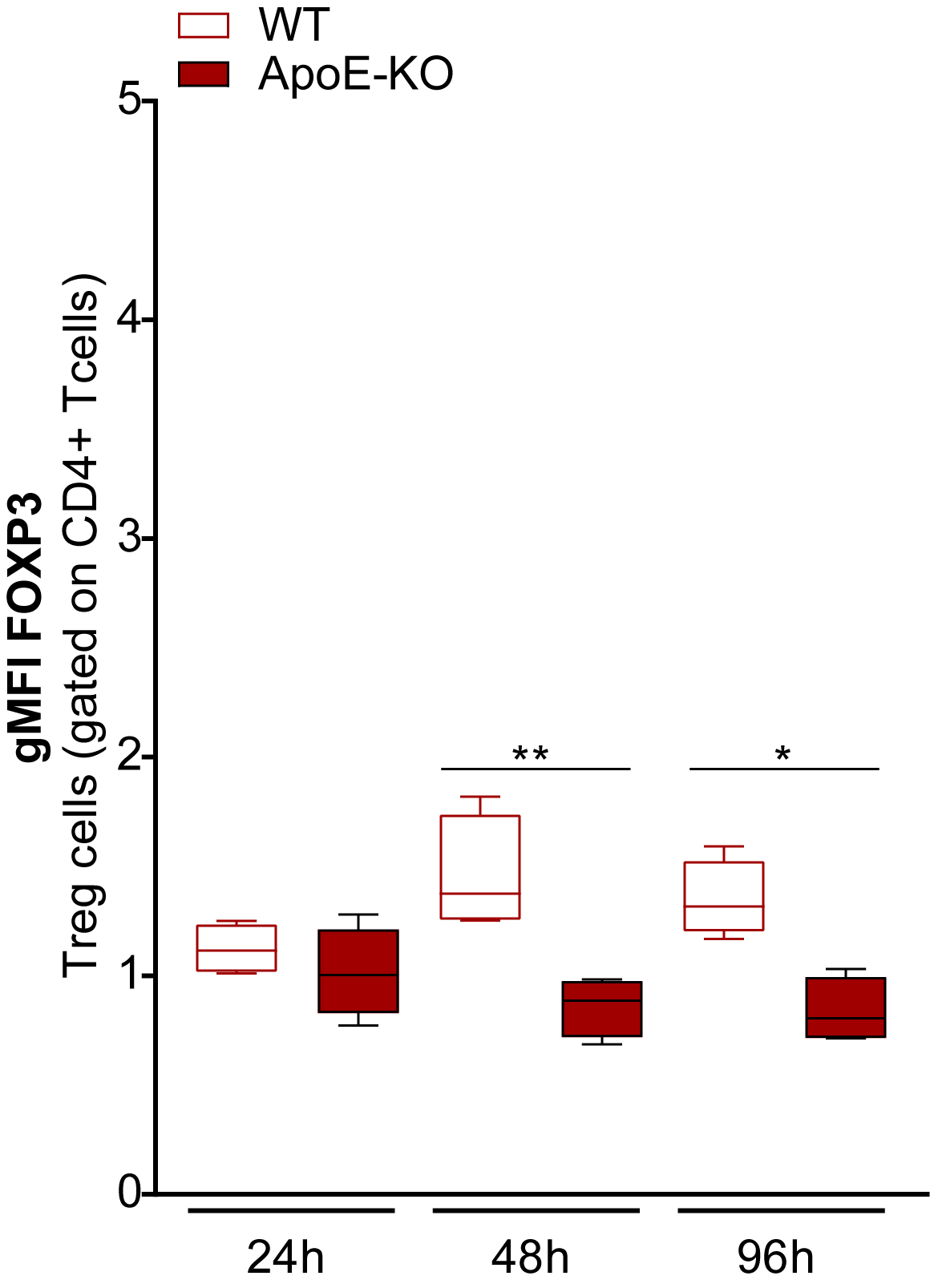

Supplement: Supplementary Figure 4 — The bar graphs represent gMFI FOXP3 in Tregs analyzed by flow cytometry. The experiments were conducted at different times: 24, 48, and 96 h, by culturing CD4+ T cells in presence of IL2 (100 U/ml), treated or not with TGFβ (2 ng/ml) and stimulated with plate-bound anti-CD3 and anti-CD28. [file Image_4.TIF]

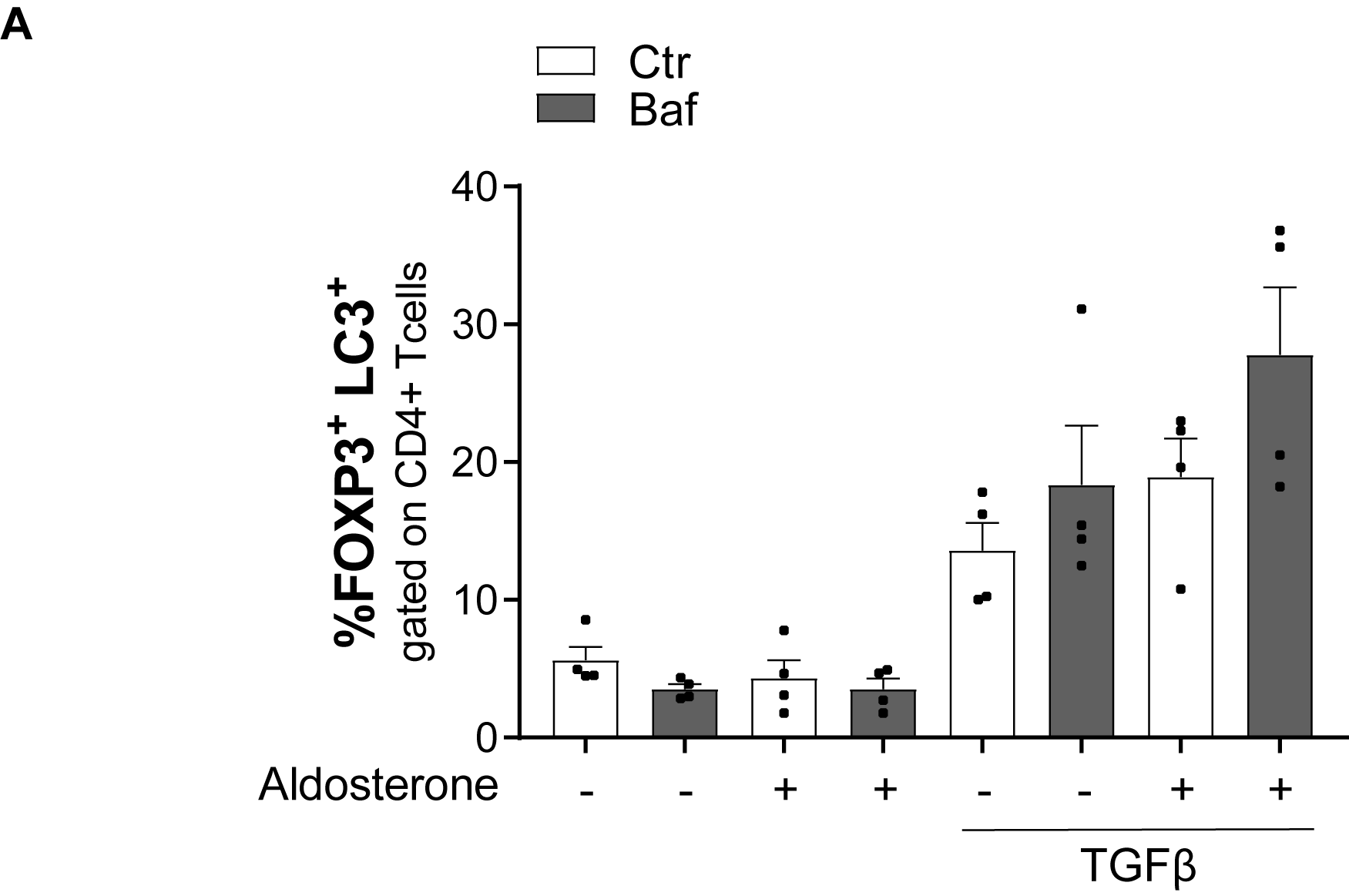

Supplement: Supplementary Figure 5 — Autophagic flux during Tregs differentiation under aldosterone treatment in wt (n = 4) mice for 96 h. Tregs were induced by culturing naïve CD4+ T cells isolated from splenocytes, in presence of IL2 (100 U/ml) treated or not with TGFβ (2 ng/ml) and stimulated with plate-bound anti-CD3 and anti-CD28. In some set of experiments aldosterone was added for 96 h. The histogram shows the frequency of Tregs-LC3-II+ (FOXP3+LC3-II+) cells gated on CD4+ T cells during 96 h of aldosterone [10−9 M] treatment. To analyze autophagic flux in some sets of experiments bafilomycin was added for during the last 4 h of stimulation. One-way Analysis of Variance (ANOVA) was performed without showing any significant differences. [file Image_5.TIF]
