# Supplementary material for: Altered Tregs Differentiation and Impaired Autophagy Correlate to Atherosclerotic Disease
Source: Front Immunol. 2020 Mar 13;11:350. doi: 10.3389/fimmu.2020.00350 (PMC7082762; doi:10.3389/fimmu.2020.00350)
Supplement: Supplementary Table 1 — The multiparameter flow cytometry analysis was used to characterize human atherosclerotic plaque. [file Table_1.docx]

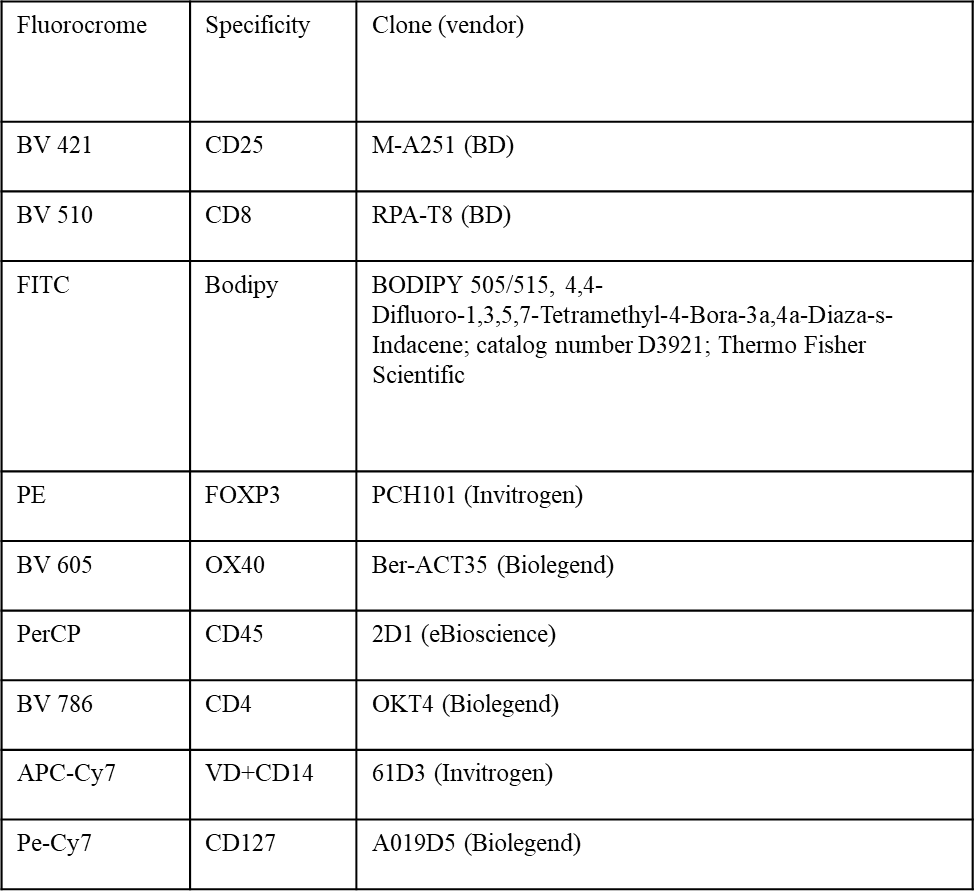


**Table S1**: The multiparameter flow cytometry analysis was used to characterize human atherosclerotic plaque
