# Supplementary material for: Altered Tregs Differentiation and Impaired Autophagy Correlate to Atherosclerotic Disease
Source: Front Immunol. 2020 Mar 13;11:350. doi: 10.3389/fimmu.2020.00350 (PMC7082762; doi:10.3389/fimmu.2020.00350)

**Table S2** The multiparameter flow cytometry analysis was used to characterize mice spleens and aortic arches


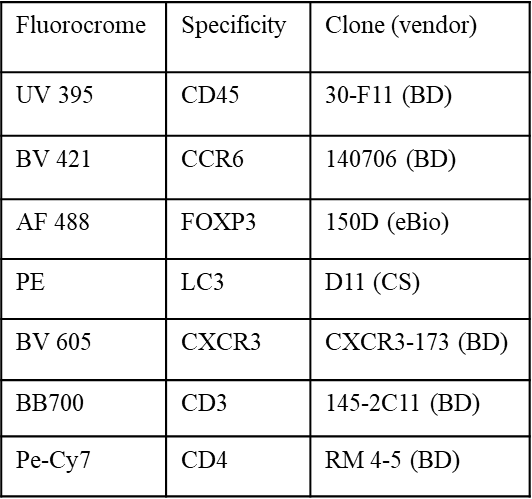

Supplement: Supplementary Table 2 — The multiparameter flow cytometry analysis was used to characterize mice spleens and aortic arches. [file Table_2.docx]
